# From biological safety to social safety: How Taiwan’s community centered prevention program controlled the COVID-19 outbreak

**DOI:** 10.7189/jogh.10.020303

**Published:** 2020-12

**Authors:** Angela Lo, Joh-Jong Huang, Cheng-Chung Chen, Frank Huang-Chih Chou, Vincent Shieh

**Affiliations:** 1 Faculty of Medicine, College of Medicine, Kaohsiung Medical University, Kaohsiung Taiwan; 2 Department of Family Medicine, Kaohsiung Medical University Chung Ho Memorial Hospital, Kaohsiung Taiwan; 3 Kaohsiung Municipal Kai-Syuan Psychiatric Hospital, Kaohsiung Taiwan; 4 Graduate Institute of Gender Education, National Kaohsiung Normal University, Kaohsiung Taiwan

## GLOBAL BIOLOGICAL DISASTER

The diversity and biological nature of global disasters are growing in frequency and severity along with rapid changes in new social patterns - such as diversity of social cultures, frequency of human movement, and convenience of communication and transportation [1]. The frequency of various types of disasters has become a worldwide concern in the 21^st^ century. Societies with a high risk of natural, human-induced, and biological disasters now have a heightened awareness and prevention efforts have necessarily become a collective responsibility of the public.

The seriousness of the biological disaster COVID-19 outbreak has required all countries to engage in extensive prevention efforts. The policies of isolation, quarantine, social-distancing and area lockdowns have been adopted by many countries. Insufficient and inaccurate information regarding COVID-19 has created a global panic which only serves to compound the problem. Prevailing anxiety has reduced social as well as economic activities with severe impact on individuals’ quality of life and employment opportunities.

Dr D Sridhar, a global health expert at the University of Edinburgh, has warned of “the Black Hole Effect” that the biological disaster may be caused by COVID-19. The concern that the media will focus on medical resources, research networks, economic benefits, national security, whereas other areas such as the restoration of interpersonal relationships, economic recovery, and social reconstruction may be neglected [2].This pandemic will seriously affect the operations of government agencies and the lives of every person.

The World Health Organization (WHO) [3,4] emphasized the value of transnational resources integration. expanding the development of epidemic prevention strategies and using comprehensive actions such as citizen governance and humanistic community-health programs. All countries need to publicize their COVID-19 circumstances, share epidemic-prevention information, and immediately conduct relevant research to develop effective epidemic-control strategies. In addition, it is critical to evaluate public-health epidemic prevention capabilities, organize required resources for disease prevention, deescalate political conflicts, address issues of government coercion, and support community health self-management [5].

## COMMUNITY-CENTERED ENGAGEMENT

Public health is an inter-disciplinary field which is informed by academic research in medical, educational, economic, political and cultural areas. One area of specialization is synthesizing this research into strategies to improve human health. Including information and resources to address epidemic prevention. During the influenza outbreak in 2017, WHO [6] published the “Pandemic Influenza Risk Management” document which highlighted different social-cultural contexts and relationship dynamics that result in people having different perceptions of risks and various trusted sources of health advice.

It is critical that medical services, epidemic specializations and community awareness efforts work together in understanding the epidemiologic triangle. This need is especially urgent in times of rising threats, such as infectious diseases, that deeply affect lifestyles and threaten human survival. Combining comprehensive risk management, all-hazards, multisectoral and multidisciplinary approaches can establish trust based on cooperation and effective communicative systems that empower community awareness. With people-centered communal engagement, we can allocate and make efficient use of local resources in carrying out the risk assessments and establishing community resilience. Thus, we can effectively eliminate the threat of infectious diseases. The major outbreak of infectious diseases deeply impacts national security, the economy, culture and vulnerabilities in the health systems. WHO listed COVID-19 community risk management as a key consideration for enhancing community resilience and practicing sustainable development in epidemic-prevention community building in the Risk Communication and Community Engagement Action Plan Guidance [7]. The COVID-19 pandemic currently poses a global threat. Without vaccines or effective drugs, measures based on biological safety, such as quarantine, sanitation, and sterilization, are the prevention strategies currently available. Taiwan employs quarantine as a biological-safety strategy to intercept infected individuals from overseas and isolate them from their communities.

## FROM BIOLOGICAL SAFETY TO SOCIAL SAFETY

To strengthen the efficiency of hospital and community disease prevention, Taiwan has established a system for epidemic-prevention indicators and risk management. Artificial intelligence technology has been employed to provide information on disease conditions and prevention and to implement a name-based system for purchasing face masks. An “epidemic- prevention name list” was established using cloud data provided by the Health Insurance Department and National Immigration Agency. In this system, patients’ travel histories can be immediately obtained using their health insurance cards. The National Health Command Center rapidly compiles epidemic information and feedback from communities. The Center organizes and enforces the Local Government Self-Quarantine and Isolation Care Service Plan to ensure that citizens are supported in daily living, mental health, and medical services [8]. It also implements community- oriented quarantine and isolation measures [9]. Nevertheless, when addressing the threat of COVID-19 community spread, epidemic-prevention measures have mainly emphasized expertise in disaster management, whereas community mobilization, citizen participation, and community partnerships during biological disasters have not been stressed. Consequently, the epidemic prevention measures based on subjective community awareness and supportive mutual care systems have not been able to address changes in the epidemic situation. Hence, local clinics and communities generate social anxiety because of insufficient psychological safety.

Community prevention measures cannot be effective merely through identifying suspected cases and implementing transmission risk controls on the basis of biological safety alone. Patients with mild symptoms and those they had contact with are difficult to identify without combined biological and social epidemic-prevention standards. The COVID-19 community epidemic-prevention operation model should include the concept of social safety. This should enable basic-level clinical workers to monitor treatment and epidemic-prevention strategies with mutual support from communities. To enhance community infection prevention efficacy, both direct and indirect outreach approaches are important. Video-streaming consultations and home visits can be conducted using artificial intelligence, and community epidemic-prevention information. Smart heath equipment can be provided using cloud data platforms. Local community clinics are the foundation of the community epidemic-prevention system, Accordingly, when responding to a COVID-19 biological disaster community clinics should have a central role in identifying, treating, and maintaining citizens’ health. Biological safety is directly connected to mental and social safety. Biological safety performance indicators can be used by local clinics and community networks to enhance strategies for outbreak prevention and maintaining daily living safety.

**Figure:**
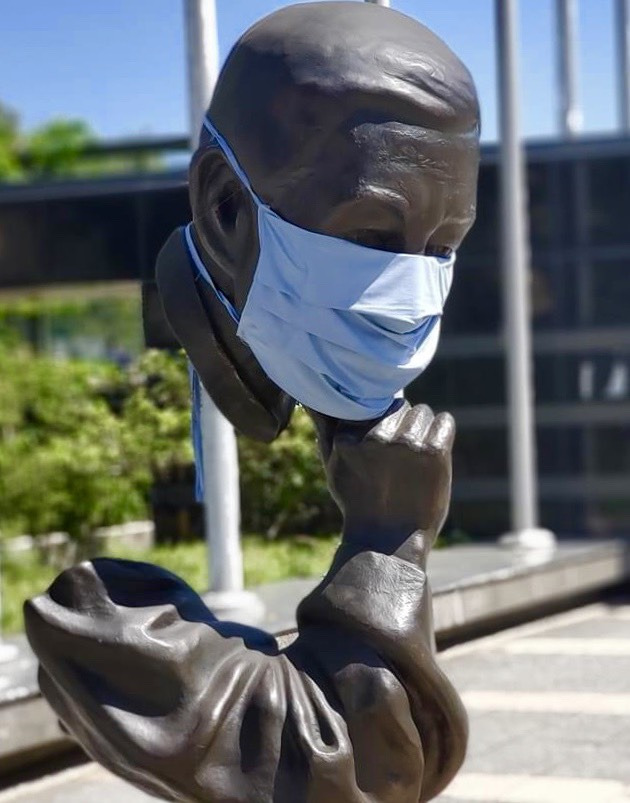
Photo: Social-safety quarantine in Taiwan.

## CONCLUSION

The leadership of President Tsai Ing-wen of Taiwan was featured in TIME Magazine [10] regarding how Taiwan prevented a major outbreak and how Taiwan responded to the various challenges. She views COVID-19 as a global disaster that requires a global prevention plan. Health safety is foundational to every country’s safety. In times of crisis, only the unity of the people will enable us to meet such challenges. Cooperation is in the nature of Taiwanese people. Thus, the government and non-governmental organizations can establish an equitable and trusted partnership, enabling the collaborative efforts of the medical network, public health systems and communities. Only with all people working together, following the lead of the medical experts, in partnership with each community, can we tackle this pandemic and enable Taiwan to become an island of resilience.

## References

[1] Lo H-WA Chen C-C Huang FH-C Chang H-T A comparison of post-traumatic stress symptoms in Chi-Chi earthquake and Morakot flood survivors. Taiwanese J Psychiatry. 2012;25:167-79.

[2] Cheng KY. Behind WHO's Wuhan Coronavirus controversy: epidemic politics and the Black Hole Effect, 2020. Available: https://english.cw.com.tw/article/article.action?id=2649. Accessed: 16 May 2020.

[3] World Health Organization. Coronavirus disease 2019 (COVID-19) Situation Report-51, 2020. Available: https://www.who.int/docs/default-source/coronaviruse/situation-reports/20200311-sitrep-51-covid-19.pdf?sfvrsn=1ba62e57_10. Accessed: 19 May 2020.

[4] World Health Organization. Coronavirus disease 2019 (COVID-19) Situation Report -57, 2020. Available: https://www.who.int/publications-detail/risk-communication-and-community-engagement-(rcce)-action-plan-guidance. Accessed: 19 May 2020.

[5] Gostin LO Hodge JG US emergency legal responses to Novel Coronavirus: balancing public health and civil liberties. JAMA. 2020;323:1131-2. 10.1001/jama.2020.2025 32207808

[6] World Health Organization. Pandemic Influenza Risk Management: A WHO guide to inform & harmonize national & international pandemic preparedness and response. Available: https://www.who.int/influenza/preparedness/pandemic/influenza_risk_management/en/. Accessed: 22 May 2020.

[7] World Health Organization. Critical preparedness, readiness and response actions for COVID-19, 2020. Available: https://www.who.int/publications-detail/critical-preparedness-readiness-and-response-actions-for-covid-19. Accessed: 16 May 2020.

[8] Taiwan Centers of Disease Control. Principles of response and treatment for severe special infectious pneumonia grass-roots clinics, 2020. Available: https://www.cdc.gov.tw/File/Get/ojEYtdWbGUoESC7GSTSNfQ. Accessed: 22 May 2020.

[9] Taiwan Centers of Disease Control. Clinic infection control new coronavirus (2019-nCOV) Q & A, 2020. Available: https://www.cdc.gov.tw/File/Get/XhEUi4Y5auEZ9mfHc0PoDg. Accessed: 28 April 2020.

[10] Tsai, Ing-wen- President of Taiwan: How my country prevented a major outbreak of COVID-19.2020 Available: https://buzzorange.com/2020/04/17/time-article-written-by-president-of-taiwan/. Accessed: 24 April 2020.

